# The role of ‘satellite crash training’ in capacity building for migration health out of Europe: István Szilárd

**DOI:** 10.1093/eurpub/ckac130.152

**Published:** 2022-10-25

**Authors:** I Szilárd, L Emődy, L Hárdy, E Marek, Z Katz, C Jaksa, I Kiss

**Affiliations:** 1 Chair of Migration Health, University of Pécs Medical School, Pécs, Hungary; 2 Institute of Medical Microbiology, University of Pécs Clinical Centre, Pécs, Hungary; 3 Management of the Foundation, Cordelia Foundation, Budapest, Hungary; 4 Institute of Public Health Medicine, University of Pécs Medical School, Pécs, Hungary

## Abstract

**Background:**

In spite of the significant shortage of migration health professionals in and out of Europe, higher education institutions are not really likely to accept WHO repeated calls for developing/ strengthening ‘migrant sensitive’ health care. Following the Syrian crisis in 2015/16, and now in Ukraine, millions of refugees have left their home country.

**Objectives:**

University of Pécs Medical School (UPMS) - based on its broad experience in migration health training -, has developed a ‘crash training’ package, easy to implement in other higher education institututions.

**Results:**

Within the frame of the program of the Hungarian Ministry of Foreign Affairs aiming to increase the migration health capacity in the Jordanian Kingdom, UPMS has established a bilateral cooperation with the Jordanian University in Amman and has offered to implement and monitor a seven-day satellite crash training on migration health.

- The program was developed in a form of ‘problem-based learning’, aiming to strengthen the self-activity of the students, while solving the task: how to establish a refugee camp addressing the challenge of high and rapid influx of migrants from the region.

- The necessary theoretical background provided by the expert team was set up around the territories as follows: international guidelines and experiences, epidemiological and public health challenges, the role of cultural competence, mental health aspects including the need for ‘helping the helpers’ as well.

**Conclusions:**

The program included a pre- and post-test component, aiming to monitor the change in knowledge, attitude, and commitment. Detailed results will be introduced during the presentation.

**Key messages:**

- Health assistance of migrants is a key public health as well as economnical interest.
- Experience of institutions on the field of migration health shoulod be utilzed globally.

